# Beneficial Effects of Estrogen in a Mouse Model of Cerebrovascular Insufficiency

**DOI:** 10.1371/journal.pone.0005159

**Published:** 2009-04-09

**Authors:** Naohito Kitamura, Runa Araya, Moeko Kudoh, Haruo Kishida, Tetsuya Kimura, Miyuki Murayama, Akihiko Takashima, Yuriko Sakamaki, Tsutomu Hashikawa, Shingo Ito, Sumio Ohtsuki, Tetsuya Terasaki, Jürgen Wess, Masahisa Yamada

**Affiliations:** 1 Yamada Research Unit, RIKEN Brain Science Institute, Saitama, Japan; 2 Laboratory for Alzheimer's Diseases, RIKEN Brain Science Institute, Saitama, Japan; 3 Research Resource Center, RIKEN Brain Science Institute, Saitama, Japan; 4 Department of Molecular Biopharmacy and Genetics, Tohoku University, Sendai, Japan; 5 Laboratory of Bioorganic Chemistry, National Institute of Diabetes and Digestive and Kidney Diseases, Bethesda, Maryland, United States of America; National Institutes of Health, United States of America

## Abstract

**Background:**

The M_5_ muscarinic acetylcholine receptor is known to play a crucial role in mediating acetylcholine dependent dilation of cerebral blood vessels. Previously, we reported that male M_5_ muscarinic acetylcholine knockout mice (*M5R* ^−/−^ mice) suffer from a constitutive constriction of cerebral arteries, reduced cerebral blood flow, dendritic atrophy, and short-term memory loss, without necrosis and/or inflammation in the brain.

**Methodology/Principal Findings:**

We employed the Magnetic Resonance Angiography to study the area of the basilar artery in male and female *M5R* ^−/−^ mice. Here we show that female *M5R* ^−/−^ mice did not show the reduction in vascular area observed in male *M5R* ^−/−^ mice. However, ovariectomized female *M5R* ^−/−^ mice displayed phenotypic changes similar to male *M5R* ^−/−^ mice, strongly suggesting that estrogen plays a key role in the observed gender differences. We found that 17β-estradiol (E2) induced nitric oxide release and ERK activation in a conditional immortalized mouse brain cerebrovascular endothelial cell line. Agonists of ERα, ERβ, and GPR30 promoted ERK activation in this cell line. Moreover, *in vivo* magnetic resonance imaging studies showed that the cross section of the basilar artery was restored to normal in male *M5R* ^−/−^ mice treated with E2. Treatment with E2 also improved the performance of male *M5R* ^−/−^ mice in a cognitive test and reduced the atrophy of neural dendrites in the cerebral cortex and hippocampus. *M5R* ^−/−^ mice also showed astrocyte swelling in cortex and hippocampus using the three-dimensional reconstruction of electron microscope images. This phenotype was reversed by E2 treatment, similar to the observed deficits in dendrite morphology and the number of synapses.

**Conclusions/Significance:**

Our findings indicate that *M5R* ^−/−^ mice represent an excellent novel model system to study the beneficial effects of estrogen on cerebrovascular function and cognition. E2 may offer new therapeutic perspectives for the treatment of cerebrovascular insufficiency related memory dysfunction.

## Introduction

Cholinergic pathways have been shown to play an important role in the regulation of cerebral vascular resistance, relaxation and contraction of blood vessels, and regional blood flow [1]–[3]. It is well known that acetylcholine (Ach) is a powerful dilator of most vascular beds and that this activity is mediated by endothelial muscarinic Ach receptors triggering the release of the actual vasorelaxing agent, nitric oxide (NO) [4]–[8]. In a previous study, we demonstrated that M_5_ receptors (M5R) mediate Ach-induced relaxation of cerebral but not of peripheral blood vessels [9], consistent with immunohistochemical [10] and pharmacological studies [11], [12].

Ach binding to M5Rs leads to the activation of G proteins of the Gq family which in turns triggers increases in intracellular calcium and inositol 1,4,5-triphosphate levels [13]–[16] and eventually the activation of endothelial NO synthase (eNOS) and the production of NO [12]. Therefore, the constitutive constriction of cerebral arteries in male *M5R^−/−^* mice that we observed in a previous study [17] is most likely due to the lack of Ach-mediated NO release.

We previously reported that male M5R knockout (*M5R^−/−^*) mice show a constitutive decrease in the diameter of the basilar artery and of middle cerebral arterioles, a reduction in cerebral blood flow (CBF), and impaired autoregulation of CBF, as compared to wildtype littermates [17]. Male *M5R^−/−^* mice also displayed dendritic atrophy in the cerebral cortex and hippocampus and impaired function of CA3 hippocampal pyramidal cells, probably secondary to cerebrovascular insufficiency [17]. Moreover, deficits in short-term memory (Y-maze) and cognitive behavior (object recognition) were evident in male *M5R^−/−^* mice, consistent with impaired hippocampal function [17]. These data suggest that male *M5R^−/−^* mice are a suitable model for evaluating the effect of decreased CBF on neurologic function. Numerous animal models of chronic cerebral hypoperfusion have been generated by surgical methods. For example, one of these models showed white matter lesions, glial activation, and spatial memory deficits, mimicking several pathological aspects of human ischemia [18], [19]. Male *M5R^−/−^* mice show also prolonged chronic cerebral hypoperfusion without necrosis in brain [17].

In the present study, we show that the phenotypic changes displayed by the male *M5R^−/−^* mice were absent in female *M5R^−/−^* mice. One possibility is that estrogens, the primary female sex hormones, protect female *M5R^−/−^* mice against the detrimental effects associated with the lack of M5Rs in males. Consistent with this notion, several studies have shown that estrogen increases CBF by stimulating the release of NO from vascular endothelial cells [20].

To test the hypothesis that estrogen protects against cerebrovascular and neuronal deficits caused by the absence of M5Rs, we conducted phenotyping studies with male *M5R^−/−^* mice bearing estrogen (E2) implants. We found that E2 treatment fully restored the diameter of the basilar artery to normal in male *M5R^−/−^* mice. Furthermore, treatment of male *M5R^−/−^* mice with estrogen also improved their cognitive performance in a Y-maze test and reduced the atrophy of neural dendrites in the cerebral cortex and hippocampus.

This study demonstrates that male *M5R^−/−^* mice, but not female *M5R^−/−^* mice, show a significantly increased number of GFAP positive cells in cortex and hippocampus, but the number of S100β positive astrocytes, a measure of the total number of astrocytes, remain unchanged. These astrocytic responses are unlikely associated with neural cells death or immunoglobulin *leakage* around cerebral blood vessels. This observation suggests that the lack of M5R in male mice leads to astrocytic swelling in cortex and hippocampus. Interestingly, E2 treatment of male *M5R^−/−^* mice completely restored normal GFAP expression levels in hippocampus. These findings indicate that *M5R^−/−^* mice represent an excellent novel model system to study the beneficial effects of estrogen on cerebrovascular function and cognition.

## Results

### Gender difference of CBF in *M5R^−/−^* mice

**Figure 1:**
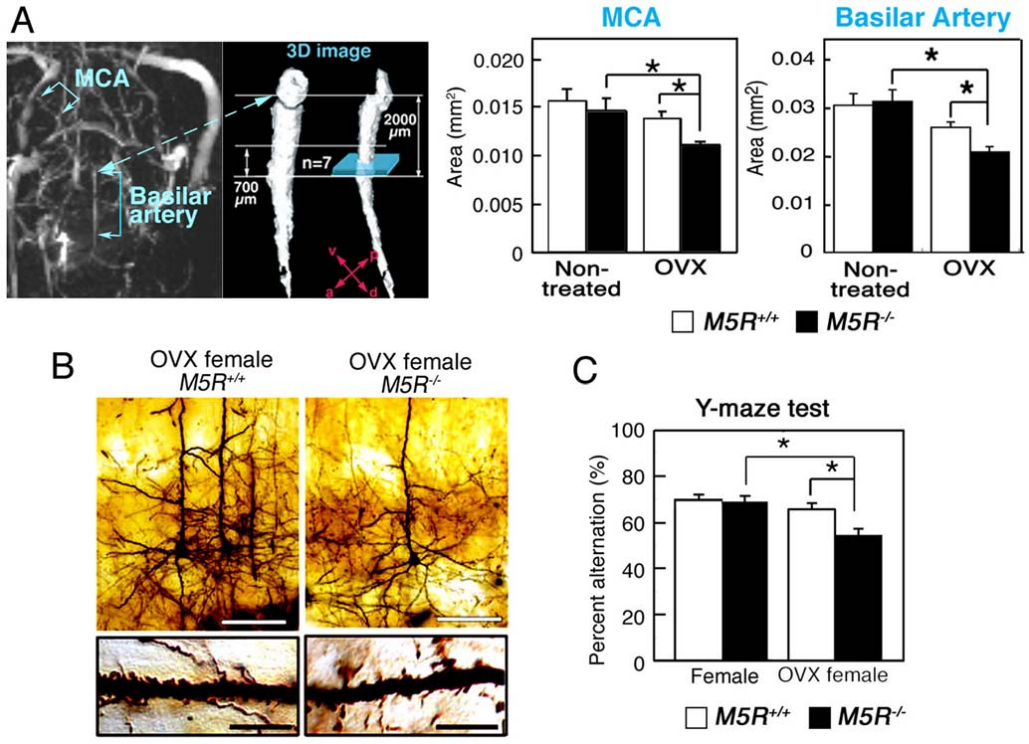
Gender-specific phenotypic differences displayed by *M5R^−/−^* mice. (A) First, rapid scanning for the basilar artery provide the each portion of artery (left image; 3D angiograms of the basilar artery from female *M5R^+/+^* mice). Second, the vascular area of each portion of the basilar artery was measured by the high resolution imaging within 30 min on a perpendicular section of the vessel (the site where the basilar artery enters the circle of Wills), and mean vascular area was determined as the average of the 7 segments on the measured area of each 100 µm intervals (right image). Ovariectomies were performed 4 months after birth using standard surgical procedures. To synchronize menstrual cycle length, we injected pregnant mare serum gonadotropin (PMSG)/human chorionic gonadotropin (hCG) into female mice by IP injection before E2 treatment (see Materials and Methods section). MRA was carried out 2 weeks after OVX surgery and two days after PMSG/hCG treatment on 4 month-old female *M5R^+/+^* (n = 11) and *M5R^−/−^* (n = 12) mice. Values are means±SE. *p<0.05. (B) Morphologic changes of cortical pyramidal neurons (layer V) from OVX female *M5R^−/−^* mice. Golgi staining studies revealed that cortical pyramidal neurons from OVX female *M5R^−/−^* mice showed clear signs of atrophy of the basal-dendritic tree and apical dendrites. White scale bars in upper panels, 50 µm; black scale bars in lower panels, 10 µm. (C) Performance of female *M5R^−/−^* and *M5R^+/+^*mice, as well as OVX *M5R^−/−^* and OVX *M5R^+/+^* mice, in the Y-maze spontaneous alternation task. Note that only OVX female *M5R^−/−^* mice showed a performance deficit in this test. Data are given as means±SEM; female *M5R^+/+^*, n = 11 mice; female *M5R^−/−^*, n = 12 mice OVX female *M5R^+/+^*, n = 20 mice; OVX female *M5R^−/−^*, n = 20 mice; *p<0.05.

We employed the Magnetic Resonance Angiography (MRA) based time-of flight (TOF) method to study the area of the basilar artery in male and female *M5R^+/+^* and *M5R^−/−^* mice. Ovariectomies were performed 4 month after birth using standard surgical procedures. To synchronize menstrual cycle length, we inject pregnant mare serum gonadotropin (PMSG) / human chorionic gonadotropin (hCG) into female mice by intraperitoneal injection (IP) before E2 treatment (see Material and Method section). MRA was carried out 2 week after ovariectomy (OVX) surgery and two days after PMSG/hCG treatments on 4 month-old female *M5R^+/+^* (n = 11) and *M5R^−/−^* mice (n = 12). TOF angiograms were obtained using a two-dimensional gradient-echo sequence (for additional experimental details, see Materials and methods). Three-dimensional angiograms of the MCA and the basilar artery (the segments indicated in **Figure 1A**) were obtained by using MATLAB software. Figure 1A shows that the diameter of the MCA and the basilar artery was similar in female *M5R^+/+^* and *M5R^−/−^* mice. In contrast, ovariectomized female *M5R^−/−^* mice showed a significant decrease in vascular diameter in the MCA and the basilar artery, as compared to intact female *M5R^−/−^* mice (**Figure 1A**).

For CBF measurements, we defined the branches of the MCA in the order from A1 to A3 (data not shown) and previously described in [17]. We measured the CBF of cerebral arterioles located on the cortical surface at the level of the peripheral branch of the MCA in male *M5R^+/+^* and *M5R^−/−^* mice (4-month-old) using laser-Doppler flowmeter. Under resting conditions, male *M5R^−/−^* mice showed a significantly lower (*p*<0.001) CBF than male *M5R^+/+^* mice at the A1 branch (see Supporting Information, **Figure S1**). However, female *M5R^−/−^* mice did not show any significant impairment in CBF, as compared to female *M5R^+/+^* mice (**Figure S1**). Furthermore, OVX *M5R^−/−^* mice showed impaired CBF (*p*<0.001), similar to male *M5R^−/−^* mice (**Figure S1**), suggesting that female sex hormones, such as estrogen, may be able to compensate for the impairment of cerebrovascular function caused by the lack of M5Rs in male mice.

### Neuroanatomical and cognitive phenotypes of female and OVX female *M5R^−/−^* mice

Male *M5R^−/−^* mice showed a significantly reduced number of dendritic spines in cortical and hippocampal CA3 pyramidal neurons [17]. We therefore also examined the number of dendritic spines in cortical pyramidal neurons (layer V) from female *M5R^+/+^* and *M5R^−/−^* mice. Golgi staining studies revealed that cortical pyramidal neurons from female *M5R^−/−^* mice (4-month-old) showed no clear signs of atrophy of the basal-dendritic tree and apical dendrites (**Figure 1B**). However, OVX female *M5R^−/−^* mice (4-month-old) showed a similar phenotype as male *M5R^−/−^* mice (**Figure 1B**). Immunostaining experiments were carried out 2 weeks after OVX surgery and two days after PMSG/hCG treatment on 4 month-old females. The number of spines in basal dendrites was significantly reduced in OVX female *M5R^−/−^* mice, as compared with OVX female *M5R^+/+^* mice (**Figure 1B**) (number of spines per 10 µm length of dendritic segment: OVX female *M5R^+/+^* mice, 24.4±0.3; OVX female *M5R^−/−^* mice, 18.1±0.9; means±SEM; P<0.05; n = 40 dendritic segments from 3 animals per group). We also analyzed the expression of several key neuronal receptor proteins by using western blot analysis (cerebral cortex and hippocampus). Specifically, we examined the expression levels of the NR1 [N-methyl-D-aspartate (NMDA) receptor subunit], GluR1 (AMPA receptor subunit), and GluR5 (kainate receptor subunit) glutamate receptor subunits. We did not observe any significant differences in glutamate receptor expression levels between female *M5R^−/−^* and *M5R^+/+^* mice (4-month-old) (**Figure S2**). However, OVX female *M5R^+/+^* mice (4-month-old) showed significantly reduced expression levels of NR1 and GluR5 receptors in cortex and hippocampus (*p*<0.05; **Figure S2**) and reduced expression of GluR1 in cortex, as compared to OVX female *M5R^+/+^* mice. GluR1 expression levels in hippocampus remained unaffected (**Figure S2**).

We next examined short time memory in female *M5R^−/−^* and *M5R^+/+^* mice, using the Y-maze spontaneous alternation task [17]. We found that locomotor activity did not differ significantly between the four groups (female *M5R^+/+^* mice, female *M5R^−/−^* mice, OVX female *M5R^+/+^* mice, OVX female *M5R^−/−^* mice; data not shown). The Y-maze test was carried out 2 weeks after OVX surgery and two days after PMSG/hCG treatment on 4 month-old females. In the Y-maze task, female *M5R^−/−^* mice (4-month-old) showed no significant impairments in spatial memory (**Figure 1C**). However, OVX female *M5R^−/−^* mice (4-month-old) showed impaired spontaneous alternation performance, similar to the male *M5R^−/−^* mice [17] (*p*<0.05; **Figure 1C**).

### Mitogen-activated protein kinase (MAPK) activation and NO release by a muscarinic agonist and E2 in TM-BBB cells

**Figure 2:**
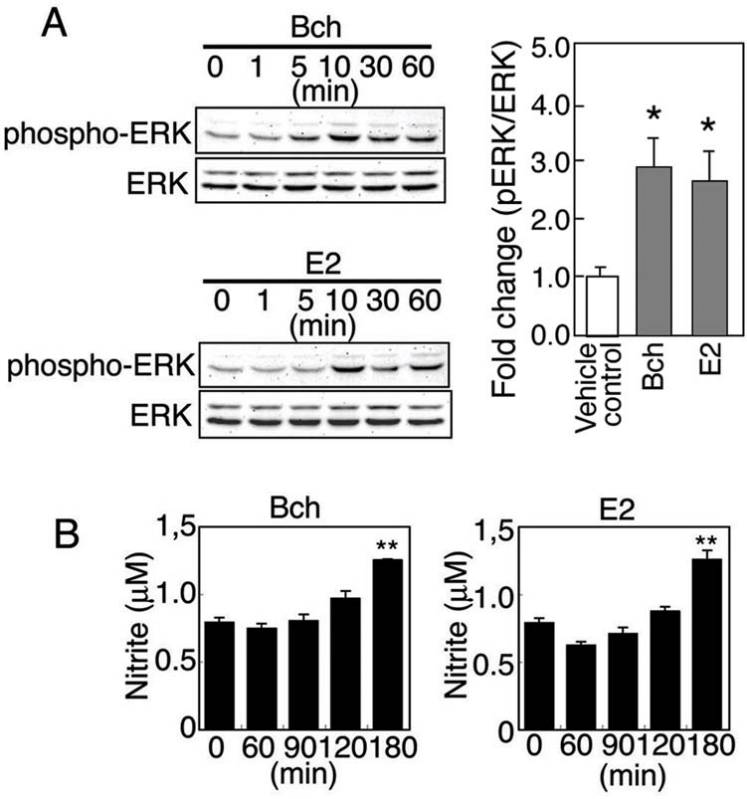
Bethanechol and E2 induce ERK phosphorylation and NO production in TM-BBB cells in a time-dependent manner. (A) TM-BBB cells were stimulated with 100 µM of the muscarinic agonist, bethanechol (Bch), or 10 nM E2 for different periods of time (1–60 min), and cell lysates were analyzed by western blotting with anti-pERK or anti-total ERK antibodies. The cumulative results of Western blot analysis are reported from three independent experiments (10 min after treatment with Bch or E2). Values are means±SEM. *p<0.05 (vs vehicle control). (B) TM-BBB cells were stimulated with 100 µM Bch or 10 nM E2 for different time periods. Subsequently, nitrite levels were measured in the culture medium. Values are means±SEM. **p<0.001 (vs vehicle control).

17β-estradiol (E2), the primary estrogen, has been reported to signal rapidly through ERK/MAP kinase [21]–[23] and PI3K/Akt [20], [24] to induce eNOS activity and NO generation in peripheral blood vessel derived cells [25], [26] and in neuroblastoma cells [27]. To examine the effect of estrogen on NO release in cerebral endothelial cells, we used the immortalized TM-BBB cell line, which is derived from mouse brain vascular endothelial cells [28] (See material and methods). TM-BBB cells were treated with bethanechol (Bch; 100 µM), a muscarinic receptor agonist, and E2 (10 nM) for 0–60 min. Bch and E2 induced the phosphorylation of ERK1/2 in a similar manner (**Figure 2A,B**). Moreover, both compounds stimulated NO production by ∼1.6-fold at the 180 min time point (**Figure 2B**). These results support the concept that both muscarinic and estrogen receptor signaling leads to enhanced NO production via activation of eNOS through ERK 1/2 pathways in TM-BBB cells [29]–[31].

**Figure 3:**
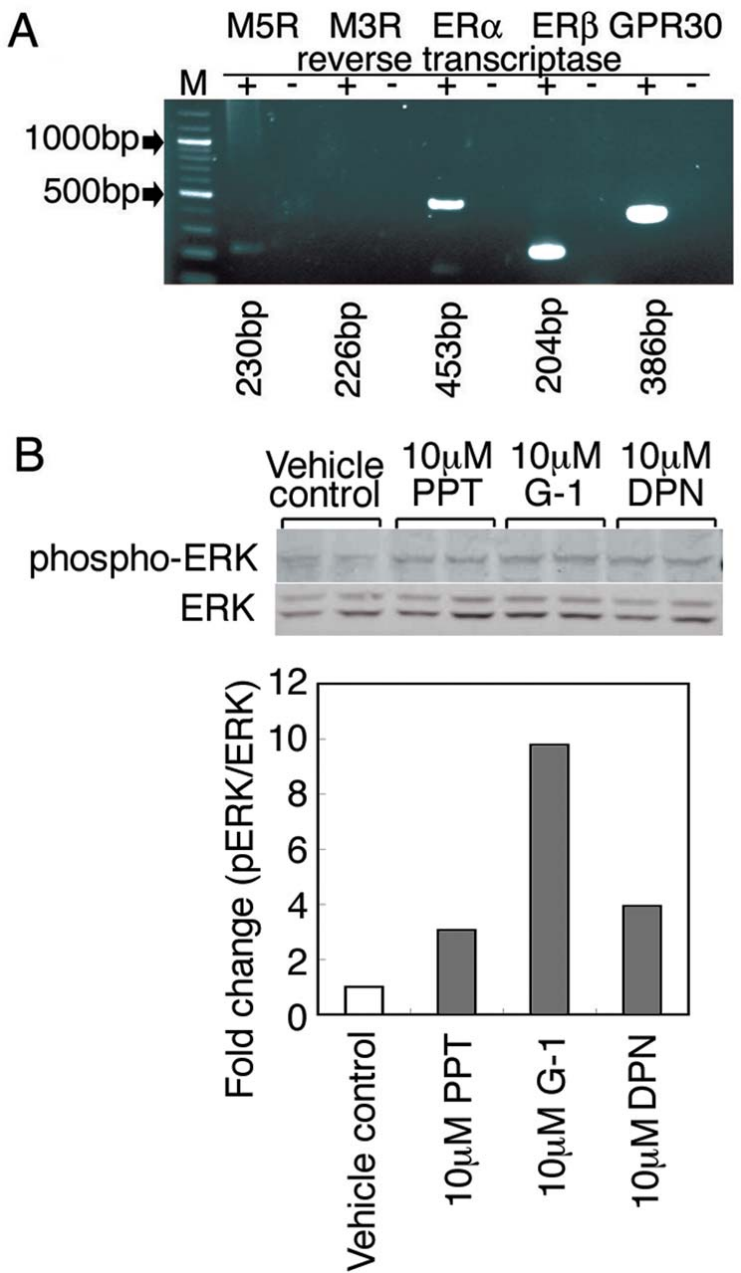
Agonists of ERα, ERβ, and GPR30 promoted ERK activation in TM-BBB cells. (A) RT-PCR analysis demonstrating the presence of transcripts of M_3_ muscarinic receptor (226 bp), M_5_ muscarinic receptor (230 bp), ERα estrogen receptor (453 bp), ERβ estrogen receptor (204 bp) and GPR30 estrogen receptor (386 bp) in TM-BBB cells. (B) TM-BBB cells were stimulated with the estrogen receptor agonists, PPT (ERα), DPN (ERβ), or G1 (GPR30) for 60 min, and cell lysates were analyzed by western blotting with anti-pERK or anti-total ERK antibodies.

RT-PCR studies showed that TM-BBB cells express M5Rs but not M3Rs which are normally found on non-cerebral arteries [32], [33], as well as ERα, ERβ and GPR30 estrogen receptors (**Figure 3A**) (See material and methods). Therefore, we also treated TM-BBB cells with selective estrogen receptor agonists, ERα (1,3,5-Tris(4-hydroxyphenyl)-4-propyl-1H-pyrazole (10 µM PPT), ERβ (10 µM DPN), and GPR30 (10 µM G1) for 60 min. Subsequently, cell lysates were prepared and analyzed by western blotting with anti-pERK or anti-total ERK antibodies. This analysis revealed the increased ERK1/2 phosphorylation mediated by 10 µM PPT, 10 µM DPN, or 10 µM G1 for 60 min (**Figure 3B**).

### Restoration of normal cerebral artery diameter by E2 in male *M5R^−/−^* and OVX female *M5R^−/−^* mice

**Figure 4:**
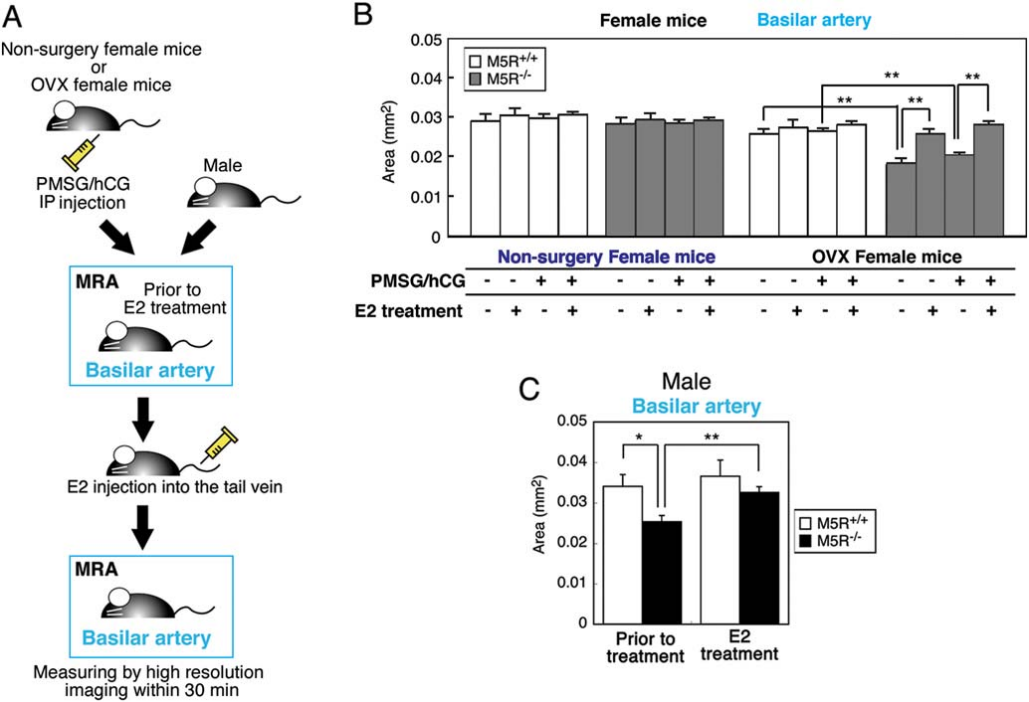
E2 injection completely restores the cross-sectional area of the basilar artery in male *M5R^−/−^* mice as well as in OVX female *M5R^−/−^* mice. (A) Schematic representation of the E2 injection and MRA monitoring schedule used. (B) Vascular area of the basilar artery in female *M5R^+/+^*, *M5R^−/−^*, OVX *M5R^+/+^*, OVX *M5R^−/−^*, and E2 injected OVX *M5R^+/+^*and *M5R* ^−/−^ mice. MRA was carried out 2 week after OVX surgery on 4 month-old female *M5R^+/+^* (n = 15) and *M5R^−/−^* (n = 15) mice. MRA was carried out 2 week after OVX surgery and two days after PMSG/hCG treatment on 4 month-old female *M5R^+/+^* (n = 14) and *M5R^−/−^* (n = 14) mice. After MRA analysis, 1 µg E2 was injected into the tail vein, followed by MRA 30 min after injection. Values are means±SE. **p<0.001. (C) Vascular area of the basilar artery in male *M5R^+/+^*and *M5R^−/−^* mice and E2 injected male *M5R^+/+^* and *M5R^−/−^* mice. 3.5–4.5 month-old male *M5R^+/+^* (n = 6) and *M5R^−/−^* (n = 10) mice were used for MRA analysis and 1 µg E2 was injected into the tail vein, followed by MRA 30 min after injection. Values are means±SEM. **p<0.001.

MRA experiments were carried out 2 weeks after OVX surgery and two days after PMSG/hCG treatment on 4 month-old females. **Figure 4B** shows that the diameter of the basilar artery was similar in female *M5R^+/+^* and *M5R^−/−^* mice (schematic representation of the E2 injection and MRA monitoring schedule, see **Figure 4A**). In contrast, OVX female *M5R^−/−^* mice showed a significant decrease in vascular diameter, as compared to intact female *M5R^−/−^* mice (**Figure 4B**). Moreover, treatment of OVX female *M5R^−/−^* mice with E2 (1 µg E2 was injected into the tail vein) completely prevented these deficits (**Figure 3B**). We noted that PMSG/hCG treatment did not significantly affect the diameter of the basilar artery in female mice (**Figure 4B**) and E2-treated male mice (**Figure S3B**).

As reported previously [17], the diameter of the basilar artery was significantly reduced in male *M5R^−/−^* mice, as compared to male *M5R^+/+^* mice (**Figure 4C**). Strikingly, E2 treatment (1 µg E2 was injected into the tail vein) of male *M5R^−/−^* mice led to an increase in the diameter of the basilar artery, similar to that observed with control male *M5R^+/+^* mice (**Figure 4C**). On the other hand, in male *M5R^+/+^* mice, E2 injection had no significant effect on vascular diameter (**Figure 4C**).

### Normalization of neurite atrophy and cognitive function by E2 treatment of male *M5R^−/−^* mice

**Figure 5:**
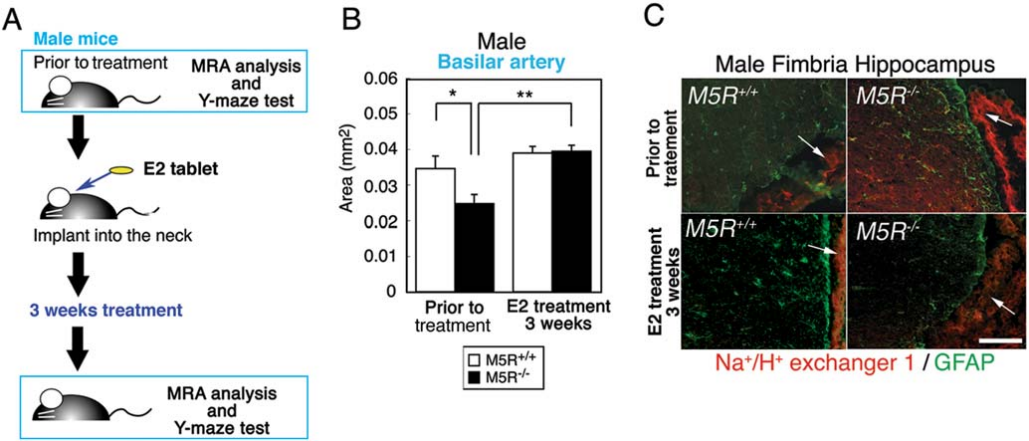
Chronic E2 treatment rescues cerebrovascular deficits in male *M5R^−/−^* mice. (A) A schematic representation schedule of E2 tablet implantation and MRA monitoring. (B) Chronic E2 treatment completely rescues cerebrovascular deficits in 3.5 month-old male *M5R^−/−^* mice. After MRA, an E2 tablet (0.1 mg / 21 days release) was implanted into the neck of each mouse, followed by MRA testing 3 weeks after the start of E2 administration. (C) The increased NHE1 expression levels in male *M5R^−/−^* mice. Na^+^/H^+^ exchanger isoform 1 (NHE1)is indicated by Red color. The GFAP is indicated by Green color. The site where the choroid plexus between fimbria of hippocampus and ventricle is indicated by arrows. Scale bar; 100 µm.

We next examined whether the deficits displayed by male *M5R^−/−^* mice could be overcome by chronic (3 weeks) E2 treatment (an 0.1 mg E2 pellet was implanted in the neck). As expected [34], non-treated male *M5R^−/−^* mice (4 months old mice) showed a significant decrease in the diameter of the basilar artery (**Figure 5B**) (a schematic representation schedule of E2 tablet implantation and MRA monitoring is given in **Figure 5A**). Strikingly, E2 treatment of male *M5R^−/−^* mice led to a recovery of vascular diameter, similar to that of control male *M5R^+/+^* mice (**Figure 5B**). PMSG/hCG treatment had not significant effect on the diameter of the basilar artery in male mice during transient or chronic E2 treatment experiments (**Figure S3**).

Hypoxic conditions affect the pH of cerebral blood serum. However, the collection of cerebral blood serum for pH measurements is technically very difficult in mice. The Na^+^/H^+^ exchanger isoform 1 (NHE1) is known to be essential for the maintenance and regulation of pH in cortical astrocytes [35]. NHE1 is also known to slightly detectable in cortical neurons [35]. We therefore examined NHE1 expression levels in astrocytes of fimbria hippocampus, an area enriched with GFAP positive astrocytes. If reduced CBF causes acidosis associated with hypoxia in the brain of male *M5R^−/−^* mice, NHE1 is predicted to be upregulated in cortical astrocytes [36]. We demonstrated that NHE1 immunolabeling was abundantly associated with plasma membrane structures of choroid plexus between fimbria of hippocampus and cortex (**Figure 5C**). In male *M5R^−/−^* mice, the intensity of NHE1 immunostaining was markedly increased in GFAP positive astrocytes in fimbria hippocampus (**Figure 5C**). E2 treatment of male *M5R^−/−^* mice restored normal NHE1 immunostaining, similar to that of control male *M5R^+/+^* mice (**Figure 5C**).

### Normalization of astrocytic swelling in cortex and hippocampus by E2 treatment of male *M5R^−/−^* mice

**Figure 6:**
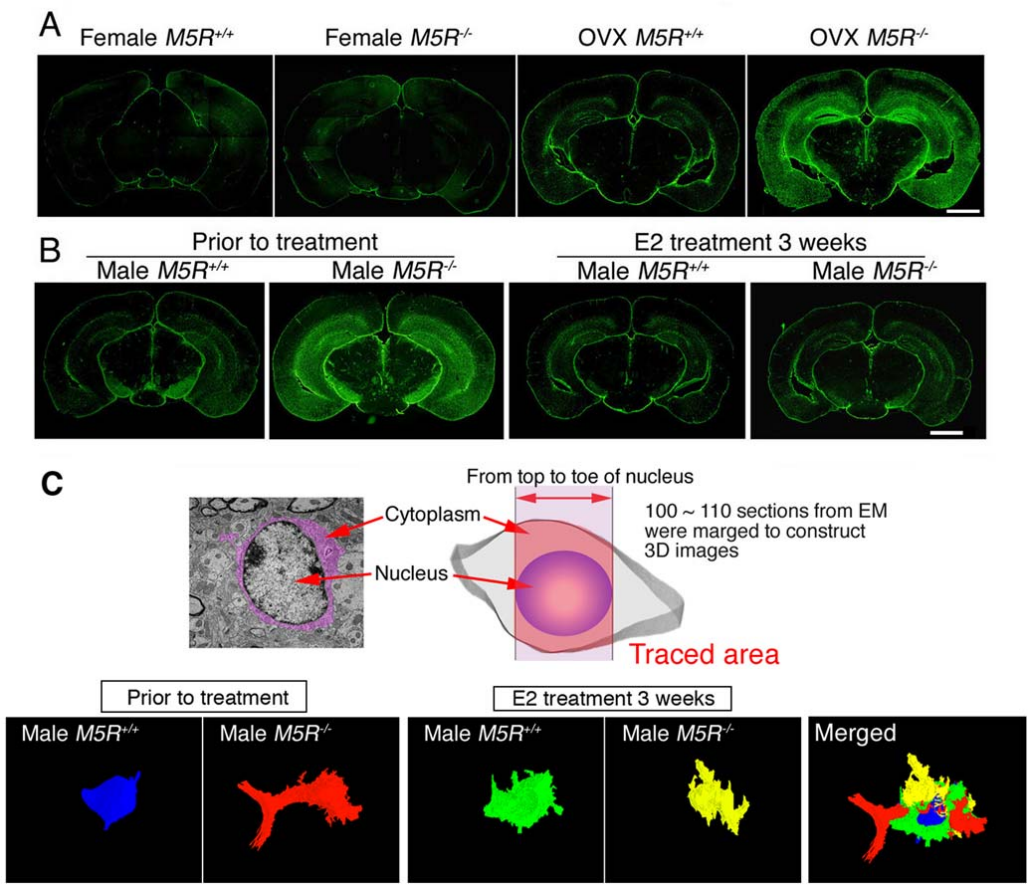
Male *M5R^−/−^* mice exhibit astrocyte swelling which is rescued by E2 treatment. (A) Female *M5R^−/−^* mice exhibit GFAP immunoreactivity similar to female *M5R^+/+^* mice in the cortex and the CA3 region of hippocampus. Immunostaining experiments were carried out 2 weeks after OVX surgery and two days after PMSG/hCG treatment on 4 month-old females. OVX *M5R^−/−^* female mice showed a significantly increased immunoreactivity of GFAP positive cells. Scale bar = 1 mm. (B) GFAP immunostaining shows astrocyte activation in cortex and hippocampus in 4-month-old male *M5R^−/−^* mice. However, E2 treatment of male *M5R^−/−^* mice restored normal GFAP expression levels. Frozen sections of the brain (coronal section) were prepared from male *M5R^−/−^* and *M5R^+/+^* mice without or after chronic E2 treatment (3 weeks, 1 µg E2 was injected into the tail vein). For details, see the legend to Fig. 3D. Scale bar = 1 mm. (C) Astrocytic swelling in the CA3 hippocampal astrocytes was visualized using three-dimensional reconstruction of electron microscope images. A total of 100–110 sections of 70 nm electron microscope images were reconstructed to visualize astrocytic shapes.

Under hypoxic conditions, changes in estrogen levels can lead to astrocytic swelling in the brain [37], [38]. On the basis of these findings, we studied the number and morphology of astrocytes in the cortex and hippocampus of E2 treated male *M5R^−/−^* and *M5R^+/+^* mice, as well as non-treated male *M5R^−/−^* and *M5R^+/+^* mice. We found that non-treated male *M5R^−/−^* mice showed a significant increase in the number of GFAP positive cells in cortex (**Figure 4A**) and hippocampus (**Figure 4A** **,** **Figure S4A–C**), but the number of S100β positive astrocytes, a measure of the total number of astrocytes, remained unchanged. Furthermore, GFAP positive astrocytes in non-treated male *M5R^−/−^* mice showed many astrocytic processes (**Figure S4C**). However, male *M5R^−/−^* mice did not show migration of microglia (Iba-1 or Mac-2 positive cells) in cortex and hippocampus (**Figure S4C**). To examine the intactness of the blood brain barrier (BBB), we injected 25 mg/kg Evans blue (EB) into the tail vain of 6-month-old male *M5R^−/−^* and *M5R^+/+^* mice, followed by visualization of auto-fluorescence of EB in cortical tissue [39]. Since EB binds to albumin, red fluorescence of EB observed in tissue indicates leakage of blood vessels. Leakage of EB in surrounding blood vessels was not detected in cortex and hippocampus of male *M5R^−/−^* and *M5R^+/+^* mice (**Figure S4E**). These data suggest that astrocytic swelling in male *M5R^−/−^* brain is unlikely to be associated with inflammation around cortical or hippocampal blood vessels. Moreover, non-treated female *M5R^−/−^* mice did not show a significant increase in the number of GFAP positive cells in cortex and hippocampus compared to female *M5R^+/+^* mice (4 months old; **Figure 6A**). However, 4-month-old OVX female *M5R^−/−^* mice showed activated GFAP positive astrocytes in cortex and hippocampus (**Figure 6A**), similar to male *M5R^−/−^* mice (**Figure 6B** **,** **Figure S4A–C**) (morphological analysis was carried out 2 weeks after OVX surgery on 4 month-old female *M5R^+/+^* and *M5R^−/−^* mice), suggesting that female sex hormones, such as estrogen, may be able to compensate for the astrocyte activation caused by the impairment of cerebrovascular function. Strikingly, E2 treatment (an 0.1 mg E2 pellet was implanted in the neck) of male *M5R^−/−^* mice restored normal GFAP expression levels (**Figure 6B** **,** **Figure S4B**). On the other hand, E2-treated male *M5R^+/+^* control mice showed similar levels of GFAP expression in cortex and hippocampus as non-treated *M5R^+/+^* mice (**Figure 6B** **,** **Figure S4A,B**).

To exclude the possibility that E2 treatment upregulates GFAP gene expression levels in male *M5R^−/−^* mice, we carried out morphological studies on CA3 hippocampal astrocytes using three-dimensional reconstruction of electron microscope images (**Figure 6C**). A total of 100–110 sections of 70 nm electron microscope images were reconstructed to visualize astrocyte shapes (see three-dimensional reconstruction of electron microscope images in Supplemental movies; non-treated male *M5R^+/+^* mice, **Movie S1**; non-treated male *M5R^−/−^* mice, **Movie S2**; E2-treated male *M5R^+/+^* mice, **Movie S3**; E2-treated male *M5R^−/−^* mice **Movie S4**). This analysis showed that the astrocyte activation leaded to the swollen astrocyte foot processes in male *M5R^−/−^* mice were markedly reduced in E2-treated male *M5R^−/−^* mice (**Figure 6C**). It is therefore unlikely that E2 treatment leads to changes in *gfap* gene expression levels in male *M5R^−/−^* mice.

### Normalization of neural morphology in cortex and hippocampus by E2 treatment of male *M5R^−/−^* mice

**Figure 7:**
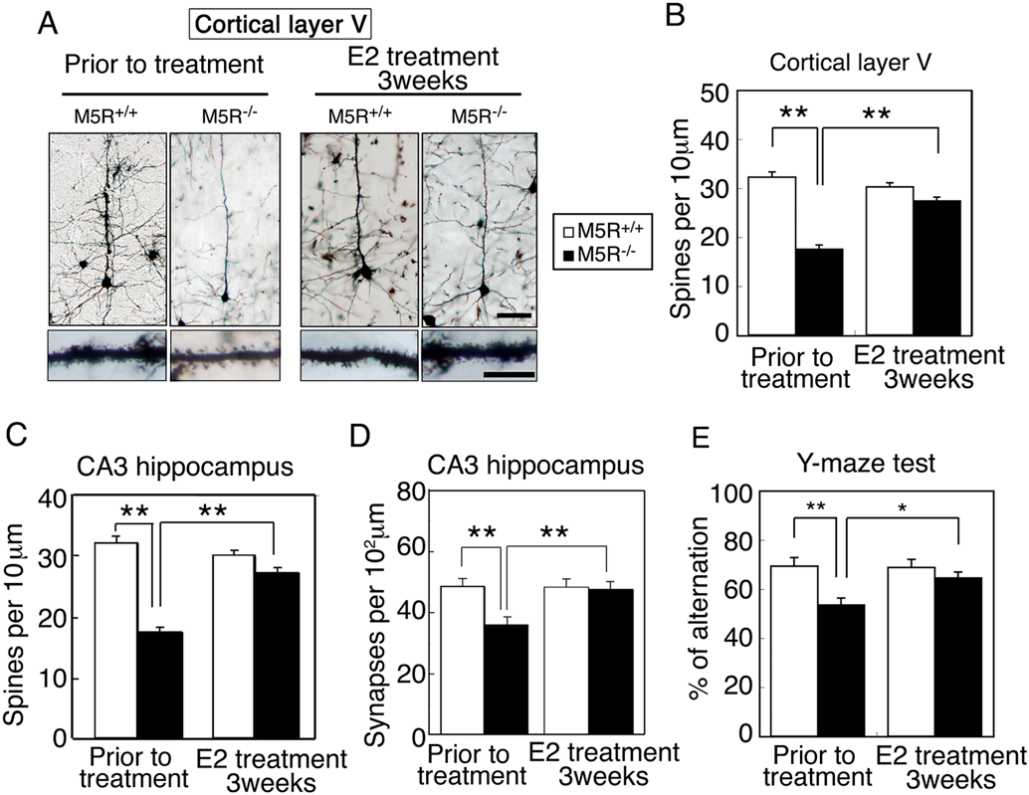
E2 restores normal morphology in cortical and hippocampal pyramidal neurons from male *M5R^−/−^* mice. (A) Morphologic changes of cortical pyramidal neurons (layer V) from male *M5R^−/−^* mice without or after chronic E2 treatment (3 weeks). Golgi staining revealed that cortical pyramidal neurons from male *M5R^−/−^* mice showed clear signs of atrophy of the basal-dendritic tree and apical dendrites. E2 treated male *M5R^−/−^* mice showed a similar morphology of spines in the basal-dendritic tree and apical dendrites as *M5R^+/+^* control mice. (B) Number of spines per 10 µm length of dendritic segment of cortical pyramidal neurons (layer V) from male *M5R^−/−^* and *M5R^+/+^* mice, and E2 treated male *M5R^−/−^* and *M5R^+/+^* mice. N = 40 dendritic segments from 5 animals per group. (C) Number of spines per 10 µm length of dendritic segment of CA3 hippocampal pyramidal neurons from male *M5R^−/−^* and *M5R^+/+^* mice, and E2 treated male *M5R^−/−^* and *M5R^+/+^* mice. Hippocampal neurons from male *M5R^−/−^* mice exhibited a significantly reduced number of dendritic spines. E2 administration also restored the number of dendritic spines in the CA3 hippocampus. N = 20 dendritic segments from 5 animals per group. Values are means±SEM. **p<0.001. (D) The number of synapses per 10^2^ µm was counted in the CA3 hippocampus by analyzing electron microscope images. A total of 100–110 sections of 70 nm electron microscope images were counted for each sample. Values are means±SEM. **p<0.001. (E) Performance of male *M5R^+/+^* and *M5R^−/−^* mice prior to and after chronic E2 treatment in a Y-maze spatial-memory test. Chronic E2 tablet (0.1 mg / 21 days release) treatment completely rescues cerebrovascular and cognitive deficits in male *M5R^−/−^* mice. All studies were carried out with 3 month-old male *M5R^+/+^* (n = 16) and *M5R^−/−^* (n = 14) mice. Values are means±SEM. *p<0.05, **p<0.001.

We examined the effect of chronic (3 weeks) E2 treatment on astrocyte morphological changes. As expected, non-treated male *M5R^−/−^* mice (4 months old) showed a significant reduction in spine number in cortical (**Figure 7A,B**) and CA3 hippocampal pyramidal neurons (**Figure 7C**), and synapse number in CA3 hippocampal pyramidal neurons (**Figure 7D**). However, after 3 weeks of E2 treatment, these morphological deficits were greatly ameliorated (**Figure 7A–D**).

We also examined the effect of chronic E2 treatment on the performance of male *M5R^−/−^* and *M5R^+/+^* mice in the Y-maze spontaneous alternation test which is commonly employed to assess spatial working memory. As reported previously, male *M5R^−/−^* mice (4 months old) showed pronounced deficits in spontaneous alternation performance (*p*<0.05; **Figure 7D**), as compared to male *M5R^+/+^* mice. After chronic E2 treatment, male *M5R^−/−^* mice did no longer show any significant impairments in this task (**Figure 7D**), indicating that E2 enhances cognitive function in male *M5R^−/−^* mice.

## Discussion

The therapeutic utility of estrogen in postischemic treatment paradigms has been studied using chronic cerebral hypoperfusion models. E2 administered after permanent middle cerebral artery occlusion or reversible middle cerebral artery occlusion reduces infarction size in ovariectomized female rats [40]–[42]. In these studies, estrogen is thought to act as a cerebral vasodilator and to protect vascular integrity. However, surgically induced animal models of cerebral hypoperfusion also lead to neuronal death and inflammation [18], [19]. It is therefore possible that the E2-mediated reduction of infarction size seen in these models may be caused by anti-inflammatory or anti-apoptotic activities of E2 [43], [44]. Thus, *M5R^−/−^* mice represent a novel genetic model of chronic cerebral hypoperfusion that can provide new insight into beneficial effects of estrogen in the absence of necrotic or inflammatory processes.

We demonstrated that male *M5R^−/−^* mice displayed a significantly increased immunostaining of GFAP positive cells in cortex and hippocampus, indicative of astrocytic activation. One possibility is that this defect is responsible for the dendritic atrophy and loss of neural functions in these regions displayed by male *M5R^−/−^* mice. These findings suggest that male *M5R^−/−^* mice represent a novel model to study the physiological and pathophysiological roles of the central cholinergic vasodilator system in regulating CBF, reactive astrocytic swelling and cognitive processes.

Strikingly, the phenotypic changes displayed by male *M5R^−/−^* mice were not observed in female *M5R^−/−^* mice, suggesting that female sex hormones might modulate CBF in female *M5R^−/−^* mice. Consistent with this notion, we observed that estrogen (E2) was able to release NO from cerebral vascular endothelial cells (NO is predicted to trigger vasodilation and increase CBF).

To test the hypothesis that estrogen may rescue the phenotypic deficits displayed by male *M5R^−/−^* mice, we implanted estrogen pellets in the dorsum of male *M5R^−/−^* mice, and initially performed MRA measurements on the basilar artery three weeks post-implantation. We found that the diameter of the basilar artery was restored to normal in male *M5R^−/−^* mice treated with estrogen. Several studies have shown that M5Rs are expressed by cerebral endothelial cells [9], [10]. Estrogen receptors (ERs) have also been shown to be expressed by vascular endothelial and smooth muscle cells [45], [46]. Estrogen binding to membrane-bound ERs can lead to the activation of G proteins such as Gs and Gq [47], [48]. M5Rs and ERs may therefore be linked to common signaling pathways in cerebral endothelial cells. The observed rapid activation of MAPKs by E2 in TM-BBB cells may therefore reflect the activation of membrane-bound ERs linked to G protein signaling pathways. Consistent with this concept, the novel membrane estrogen receptor GPR30 [29]–[31] was also detected in the TM-BBB cell line (**Figure 3A**). We found that PPT (an ERα agonist), DPN (an ERβ agonist), and G1 (a GPR30 agonist) promoted ERK activation (**Figure 3B**). Therefore, a combination of estrogen receptors, ERα, ERβ, and GPR30, may allow for sufficient ERK1/2 phosphorylation.

E2-mediated NO production has been linked to vasodilation and is thought to involve stimulation of eNOS following rapid activation of phosphatidylinositol-3 kinase (PI3K)/Akt or MAPK signaling in different vascular tissues [20], [21], [25], [49].

In the present study, we demonstrated that OVX female *M5R^−/−^* mice, in contrast to non-OVX female *M5R^−/−^* mice, suffer from a constitutive constriction of cerebral arteries, reduced CBF, dendritic atrophy (**Figure 1A**), and short-term memory loss (**Figure 1C**). We also found that E2 injection completely restored the diameter of the basilar artery in OVX female *M5R^−/−^* mice (as compared to non-OVX female *M5R^−/−^* mice). E2 partially restored the vascular area in OVX female *M5R^+/+^* mice (as compared to non-OVX female *M5R^+/+^* mice). In addition, E2 rescued the constitutive constriction of the basilar artery in male *M5R^−/−^* mice. These results suggest that estrogen (E2) can compensate for the lack of M5R-mediated vasodilation in cerebral blood vessels in *M5R^−/−^* mice. *M5R^−/−^* mice can therefore be used as novel model to examine the vasodilator effects of estrogen *in vivo*. Therefore, estrogen therapy may be clinically useful not only for female but also for male patients suffering from cerebrovascular deficits.

It is known that ischemia results in a marked reduction of tissue pH [50] and cerebral ischemia has been demonstrated to produce an intracellular acidosis in the ischemic core as well as in the ischemic penumbra [51]. For example, the NHE1 is one of the major acid extrusion mechanisms after intracellular acidosis. NHE1 catalyzes the electroneutral exchange of H^+^ and Na^+^ ions across cellular membranes, thereby regulating the pH of the cytoplasm [52], [53]. Therefore, expression of NHE1 is thought to protect cells from internal acidification and to regulate cell volume in response to hypoxia. We found significantly increased NHE1 expression levels in male *M5R^−/−^* mice (**Figure 5C**). Swollen astrocyte foot processes lead to changes in brain extracellular space volume, composition, and geometry in aged animals with severe learning impairment [49]. It is hypothesized that the degree of hippocampal learning impairment in aged animals is related to astrocyte swelling [49]. Therefore we speculated that hypoxic stimulation induced astrocyte activation might contribute to the changes of neural activity in brain of male *M5R^−/−^* mice. We found that the lack of M5Rs in male mice was associated with cortical and hippocampal astrocytic swelling and deficits in dendritic morphology. It is well known that changes in astrocyte function regulate synaptic transmission [54]–[57]. It is tempting to speculate that the astrocytic swelling phenotype observed with male *M5R^−/−^* mice is a consequence of cerebrovascular insufficiency. Strikingly, E2 treatment of male *M5R^−/−^* mice led to a reversal of the astrocytic swelling phenotype, which can occupy extra-synaptic spaces (**Figure 6C**), and restored normal numbers of dendritic spines of pyramidal neurons in cortical and hippocampal pyramidal neurons (**Figure 7A–D**). Treatment with E2 also improved the performance of male *M5R^−/−^* mice in a cognitive test (Y-maze spontaneous alternation task)(**Figure 7E**). Estrogen treatment did not affect GFAP expression levels in male *M5R^+/+^* mice, as studied by western blotting analysis (**Figure S4B**). It is therefore unlikely that E2 acts on astrocytes directly to reduce GFAP immunoreactivity. GFAP expression levels were strikingly correlated with NHE1 expression levels in astrocytes, probably as a consequence of the hypoxic state of the brain of male *M5R^−/−^* mice. Our findings support the idea that E2 regulates important neuroprotective mechanisms *in vivo* by acting directly on endothelial cells and/or through glial cell intermediaries. Estrogen has been demonstrated to have numerous effects on glial cells and neurons. Besides affecting CBF by acting on vascular ERs, E2 may protect against neuronal damage through actions on glial cell activation [36], [37]. Furthermore, animal studies suggest that E2 has neuroprotective effects against excito-toxicity [58], oxidative damage [59], and cerebral ischemia [60]. Our observations support the concept that the beneficial effects of estrogen on neural function observed in the present study are due to cerebral vasodilation controlling astrocyte activation as well as a direct neuroprotective effect. Clearly, additional studies are needed to examine the causal relationship between the cerebrovascular, neuromorphological, and behavioral deficits displayed by male *M5R^−/−^* mice and to further explore the mechanisms underlying the ability of estrogen to reverse these phenotypes.

## Materials and Methods

### Animals

*M5R^−/−^* mice were produced as described previously [9]. The average menstrual cycle length is 4 days in mice. To synchronize menstrual cycle length, we injected pregnant mare serum gonadotropin (PMSG)/human chorionic gonadotropin (hCG) into female mice by intraperitoneal (IP) injection before E2 treatment. Female mice were primed with an IP injection of 7.5 IU of PMSG and 7.5 IU of hCG to induce ovulation. After two days, female mice were subjected to the experiments. Animal experiments were approved by the Animal Experiment Committee of the RIKEN Brain Science Institute.

### Measurement of CBF using a laser Doppler flowmeter

CBF measurements were performed as previously described [17]. The branches of the MCA were defined in the order from A1 to A3 [17]. A probe with a diameter of 0.5 mm was attached to the point of divergence of the MCA, and CBF was measured continuously in the parietal lobe using a laser Doppler flowmeter (ALF 21, Advance Co., Ltd, Tokyo), in conjunction with a PowerLab system (AD Instruments, CA, USA).

### MRA analysis

MRA was performed as previously described [17], with minor modifications. Mice were anesthetized with pentobarbital and subjected to micro-MRI scans using a vertical bore 9.4 T Bruker AVANCE 400WB imaging spectrometer with a 250 mT/m actively shielded microimaging gradient insert (Bruker BioSpin GmbH, Ettlingen, Germany) [17]. A 25-mm resonator was used for signal excitation and detection. The depth of anesthesia was monitored with a breathing sensor, and was maintained with 0.5 to 1.5% isoflurane in air (2 l/min flow rate). Two-dimensional horizontal MRA images were acquired by using a method derived from gradient-echo pulse sequence with flow compensation. The scans were performed with the following imaging parameters: TR = 50.0 ms; TE = 5.0 ms; flip angle = 25°; matrix = 256·256; field of view = 2·2 cm^2^; number of slices = 40; slice thickness = 0.05 mm; and total imaging time = 51 min (10 averages). Angiograms were obtained by generating maximum intensity projections using Paravision software (Bruker BioSpin GmbH). Basilar arterial vascular area was measured on the perpendicular section of the vessel using OSIRIX software, and mean vascular dimension was determined as the average of 7 segments in the measured area (Fig. 3A).

### Ovariectomy and E2 implantation

Ovariectomies were performed 4 months after birth using standard surgical procedures. MRA was carried out 2 weeks after OVX surgery on 4 month-old female *M5R^+/+^* and *M5R^−/−^* mice. For transient E2 treatment experiments, 1 µg E2 was injected into the tail vein. For the chronic administration (3 weeks) of estrogen pellets (steady-release pellet of an 0.1 mg 17β-estradiol pellet) to adult male mice, a small incision was made in the dorsal neck region for the insertion of the hormone pellet (Innovative Research) to maintain plasma estrogen levels ranging from 250 to 260 pg/mL, and the incision was closed with a wound clip under anesthesia. The doses used in these experiments were chosen based upon their ability to generate physiological serum concentrations [19]. Estrogen was below the level of detection prior to E2 treatment of male mice.

### Cell culture

The conditionally immortalized mouse brain capillary endothelial cell line (TM-BBB) was performed as previously described [61]. The culture medium used consisted of DMEM (Gibco, Carlsbad, CA) supplemented with 15 µg/ml endothelial cell growth factor (Roche Diagnostics, Indianapolis, IN), 100 U/ml benzylpenicillin potassium, 100 µg/ml streptomycin sulfate, and 10% fetal bovine serum.

### Immunoblotting

TM-BBB cells were seeded at a density of 6.5×10^4^ cells per dish on collagen type I-coated 35-mm culture dishes (Becton Dickinson). After 48 h of culture, cells were incubated for 24 h in growth factor-free medium followed by 2 h incubation in serum-free fresh medium at 33 °C. TM-BBB cells were incubated at 33 °C with vehicle, bethanechol 100 µM (Bch: MP Biomedicals, Solon, OH) or 17-β-estradiol 10 nM (E2: Sigma Chemical Co.) as indicated. TM-BBB cells were also treated with selective activators of ERα (1,3,5-Tris(4-hydroxyphenyl)-4-propyl-1H-pyrazole; PPT, Sigma), ERβ (DPN, Tocris Bioscience), and GPR30 (G1, MERCK). Cells were rinsed with ice-cold phosphate-buffered saline (PBS) containing 0.5 mM Na3VO4 and then collected in 100 µl of lysis buffer (50 mM Tris-HCl, pH 7.5, containing 150 mM NaCl, 1% Triton X-100, 1% deoxycholate, 0.2 mM Na_3_VO_4_, 1 mM EGTA, 0.4 mM EDTA, 1 mg/ml of aprotinin and leupeptin, and 0.1 mg/ml of phenylmethylsulphonyl fluoride). Lysates were centrifuged at 10,000 g for 15 min at 4°C to remove insoluble material and normalized for protein content. Equal amounts of protein (20 µg) were separated by 10% SDS–PAGE, transferred to nitrocellulose membranes (Schleicher & Schuell), probed with rabbit polyclonal phospho-p44/42 MAP kinase antibody or rabbit polyclonal p44/42 MAP kinase antibody (Cell Signaling Beverly, MA). Subsequently, the membranes were incubated with goat anti-rabbit IgG secondary antibodies (PerkinElmer Life Sciences Inc. Boston, MA).

### RT-PCR

Total RNA was extracted from tissues derived from TM-BBB using Trizol reagent (GIBCO). After DNase treatment (2 units µl^−1^; Ambion), total RNA (≈1 µg) was reverse-transcribed by using oligo (dT)16 primers and murine leukemia virus RT (Perkin–Elmer), followed by PCR amplification of a DNA segment specific for each receptor gene (PCR conditions: 98°C for 2 min; 35 cycles of 98°C for 10 sec and 55°C for 30 sec; 72°C for 5 min). For PCR amplification for the ER alpha gene, 5′-TGGCGCTCCATGGAACAC-3′ and 5′-CATCTCCAGGAGCAGGTC-3′; ER beta gene, 5′-AAAGCCAAGAGAACCAGTGGGCAC-3′ and 5′-GCCAATCATGTGCACCAGTTCCTT-3′; GPR30 gene, 5′-ATCTGGATGGCCTCAGTGTC-3′ and 5′-ACTATGTGGCCTGTCAAGGG-3′; M3 receptor gene, 5′-ACCAAGACCACAGTAGCAGTG-3′ and 5′-CTCTCTACATCCATAGTCCC-3′; M5 receptor gene, 5′-GTCTCCGTCATGACCATACTCTA-3′ and 5′-CCCGTTGTTGAGGTGCTTCTAC-3′

### Nitrite assay

NO production was determined by measuring nitrite accumulation in culture medium using the NO2/NO3 Assay Kit-FX (Dojindo Laboratories, Kumamoto, Japan) according to manufacturer's instructions. Briefly, TM-BBB cells were seeded at a density of 6×10^4^ cells per dish on collagen type I-coated 24 well plates (IWAKI, Chiba, Japan). After 48 h of culture, cells were washed twice with phenol red-free and serum-free MEM (Gibco) followed by incubation in 300 µl of the same medium containing vehicle, Bch or E2. After this incubation period, 300 µl of medium were removed from each well and centrifuged at 1,000 g for 15 min at room temperature. Supernatants (80 µl aliquots) were mixed with 20 µl of Buffer solution (pH 7.6) and 10 µl of fluorescence reagent (2,3-Diaminonaphthalene) solution, followed by incubation for 30 min at room temperature. The reaction was terminated by the addition of 30 µl of Stop solution. Fluorescence was measured using a spectrofluorometer (ARVO MX, PerkinElmer Life Sciences Inc.), with excitation and emission wavelengths set at 355 and 460 nm, respectively

### Immunohistochemistry and Golgi staining

Frozen cryosections (15 µm) were treated with 3% H_2_O_2_, incubated with blocking buffer (PBS containing 0.01% Triton and 1.5% normal goat serum), and incubated overnight at 4°C with primary antibodies. Sections were incubated with secondary antibody in blocking buffer for 1 h at room temperature. The primary antibodies used were anti-GFAP Ab (DAKO), anti-Iba-1 (Wako, Japan), and anti-Mac-2 (American Type Culture Collection, USA). Secondary antibodies were alexa fluor 546 goat anti-mouse IgG (Molecular Probes) and alexa fluor 488 goat anti-rabbit IgG (Molecular Probes). Sections were counterstained with Hoechst (CALBIOCHEM). Golgi staining was performed essentially as described previously [17]

### Electron microscopy

For electronmicroscopic observation of astrocytes in the CA3 region of the hippocampus, a *M5R^−/−^* mouse and a wild-type control mouse were fixed 2.5% glutaraldehyde and 4% paraformaldehyde. After fixation, the tissue samples were postfixed with 1% (w/v) osmium tetroxide in 0.1 M PB, dehydrated in a graded ethanol series, and embedded in epoxy resin (EPON812, TAAB, Aldermaston, UK). For electron microscopic observations, the brain was sectioned into a series of 100–110 sections (70 nm thick) with an ultramicrotome (EM UC6, Leica, Heidelberg, Germany). Ultrathin sections were stained with uranyl acetate and lead citrate. Electron micrographs recorded on imaging plates through a JEM-1200EX electron microscope (JEOL DATUM LTD., Tokyo, Japan) were scanned and digitized by an FDL 5000 imaging system (Fuji Photofilm, Tokyo, Japan). To reconstruct 3D images of astrocytes, each series was aligned using sEM Align software and edges of astrocytic cytoplasm were traced using IGL Trace software based on ref. [62] (freely available at http:// synapse-web.org/tools/reconstruct/reconstruct.stm).

### Y-maze test

The Y-maze task was performed essentially as described previously [17].

### Statistical Analysis

Data are expressed as mean±SEM. Statistical comparisons of CBF, nitrite, and MRA data were performed using Student's *t*-test or ANOVA. *p*<0.05 was considered to be statistically different.

## Supporting Information

Figure S1 Gender-specific phenotypic differences in CBF displayed by M5R−/− mice. CBF was measured in the middle cerebral artery and arterioles (MCA). MCA branches were defined in the order from A1 to A3 for classification scheme, described [17]. CBF was measured in the A1 area by laser-Doppler flowmetry. Male M5R−/− mice showed significantly reduced CBF, as compared to male M5R+/+ mice. In contrast, female M5R+/+ and M5R−/− mice displayed similar CBF. OVX female M5R−/− mice showed reduced CBF, similar to male M5R−/− mice. Data are expressed as CBF relative to M5R+/+ (white bars). OVX mice were used for CBF measurements 4 weeks after surgery performed on 4-month-old female M5R−/− and M5R+/+ mice. The numbers given in parentheses under the bars indicate the number of independent experiments (mice). Data are means±SEM. **p<0.001 (vs M5R+/+ mice).
(11.24 MB TIF

Figure S2 Gender-specific phenotypic differences displayed by female M5R−/− mice. Relative expression levels, determined by Western blot analysis, of cortical (top panel) and hippocampal (lower panel) glutamate receptor subunits in female M5R−/− and M5R+/+ ( = 100%) mice, and OVX M5R−/− and OVX M5R+/+ ( = 100%) mice. The numbers given in parentheses underneath the bars indicate the number of independent experiments (mice). Data represent means±SEM; *p<0.05; **p<0.001.
(11.24 MB TIF)

Figure S3 PMSG/hCG treatment did not significantly affect the diameter of the basilar artery in male mice. (A) Experimental schedule for PMSG/hCG/E2 treatment experiments. (B) PMSG/hCG treatment did not affect the diameter of the basilar artery during transient or chronic E2 treatment experiments. All studies were carried out with 3 month-old male M5R+/+ and M5R−/− mice (n = 12 per group). Data represent means±SEM; *p<0.05; **p<0.001.
(9.00 MB TIF)

Figure S4 Male M5R−/− mice exhibit astrocyte activation without migration of microglia. Frozen sections of cerebral cortex and hippocampus were prepared from 4-month-old male M5R+/+ and M5R−/− mice. An E2 tablet (0.1 mg/21 days release) was implanted into neck of each mouse. (A) GFAP immunostaining signals in the hippocampal CA3 region from E2-treated male M5R−/− and M5R+/+ mice vs. non-treated male M5R−/− mice. Scale bar = 200 µm. (B) E2 treatment of male M5R−/− mice restored wild-type-like GFAP protein expression levels in the hippocampus, as studied by western blotting analysis. E2-treated male M5R+/+ mice showed similar GFAP protein expression levels as non-treated male M5R+/+ mice. Data are means±SEM (n = 8 per group). *p<0.05 (vs M5R+/+ mice). (C) Male M5R−/− mice showed a significantly increased number of GFAP positive cells in the CA3 region of the hippocampus. However, the number of S100β positive astrocytes, a measure of the total number of astrocytes, remained unchanged. Five hippocampal CA3 sections from 5 animals per group were analyzed. Values are means±SE. **p<0.001. (D) Vimentin and neurocan, markers for injured astrocytes, did not show any increase in immunoreactivity in male M5R−/− mice. Scale bar, 1 mm. (E) Leakage of EB in surrounding blood vessels was not detected in cortex and hippocampus of male M5R−/− and M5R+/+ mice. Scale bar, 1 mm.
(11.24 MB TIF)

Movie S1 (0.96 MB SWF)

Movie S2 (1.90 MB SWF)

Movie S3 (2.04 MB SWF)

Movie S4 (1.85 MB SWF)
