# Supplementary figures and images for: Beneficial Effects of Estrogen in a Mouse Model of Cerebrovascular Insufficiency
Source: PLoS One. 2009 Apr 9;4(4):e5159. doi: 10.1371/journal.pone.0005159 (PMC2664330; doi:10.1371/journal.pone.0005159)

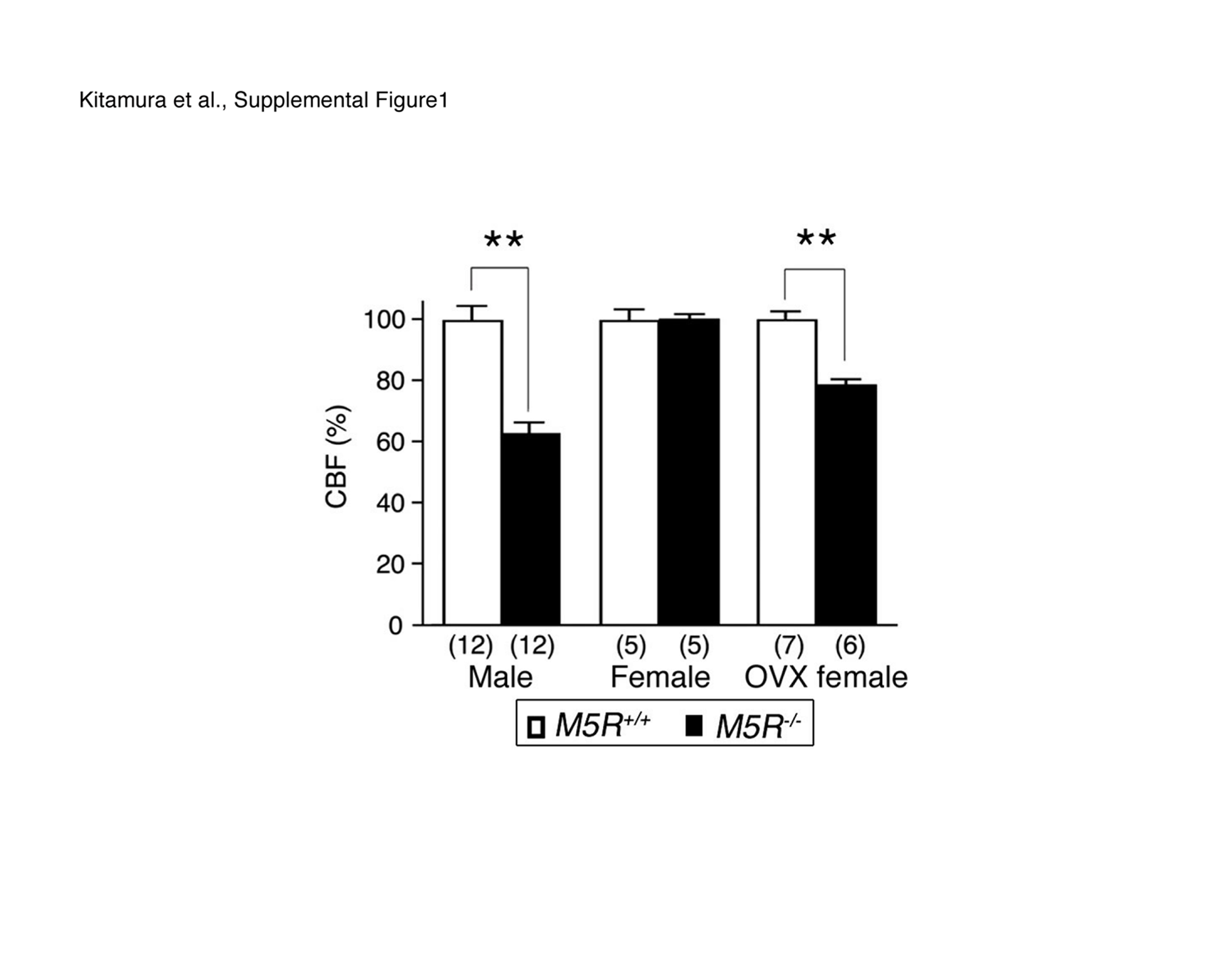

Supplement: Figure S1 — Gender-specific phenotypic differences in CBF displayed by M5R−/− mice. CBF was measured in the middle cerebral artery and arterioles (MCA). MCA branches were defined in the order from A1 to A3 for classification scheme, described [17]. CBF was measured in the A1 area by laser-Doppler flowmetry. Male M5R−/− mice showed significantly reduced CBF, as compared to male M5R+/+ mice. In contrast, female M5R+/+ and M5R−/− mice displayed similar CBF. OVX female M5R−/− mice showed reduced CBF, similar to male M5R−/− mice. Data are expressed as CBF relative to M5R+/+ (white bars). OVX mice were used for CBF measurements 4 weeks after surgery performed on 4-month-old female M5R−/− and M5R+/+ mice. The numbers given in parentheses under the bars indicate the number of independent experiments (mice). Data are means±SEM. **p<0.001 (vs M5R+/+ mice). (11.24 MB TIF [file pone.0005159.s001.tif]

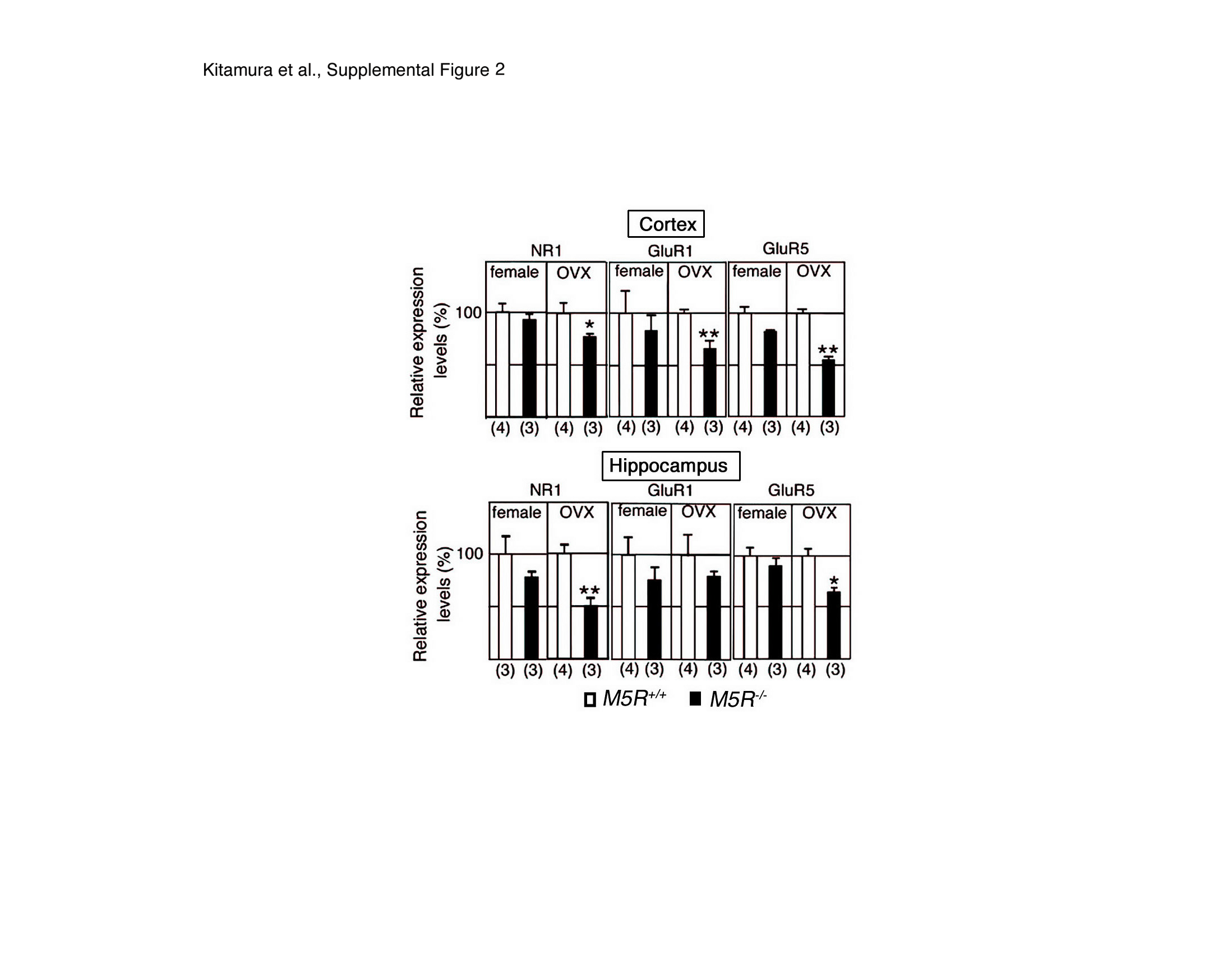

Supplement: Figure S2 — Gender-specific phenotypic differences displayed by female M5R−/− mice. Relative expression levels, determined by Western blot analysis, of cortical (top panel) and hippocampal (lower panel) glutamate receptor subunits in female M5R−/− and M5R+/+ ( = 100%) mice, and OVX M5R−/− and OVX M5R+/+ ( = 100%) mice. The numbers given in parentheses underneath the bars indicate the number of independent experiments (mice). Data represent means±SEM; *p<0.05; **p<0.001. (11.24 MB TIF) [file pone.0005159.s002.tif]

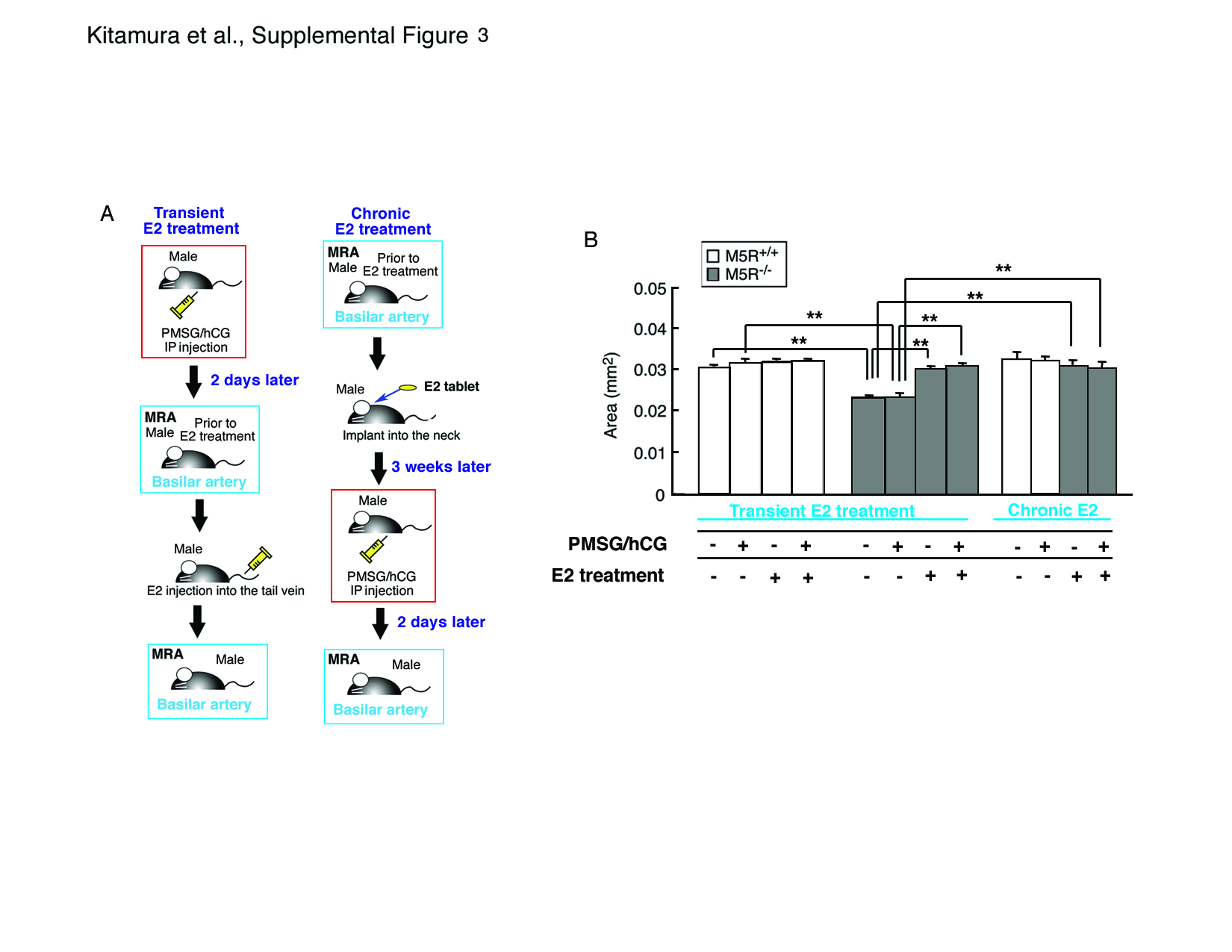

Supplement: Figure S3 — PMSG/hCG treatment did not significantly affect the diameter of the basilar artery in male mice. (A) Experimental schedule for PMSG/hCG/E2 treatment experiments. (B) PMSG/hCG treatment did not affect the diameter of the basilar artery during transient or chronic E2 treatment experiments. All studies were carried out with 3 month-old male M5R+/+ and M5R−/− mice (n = 12 per group). Data represent means±SEM; *p<0.05; **p<0.001. (9.00 MB TIF) [file pone.0005159.s003.tif]

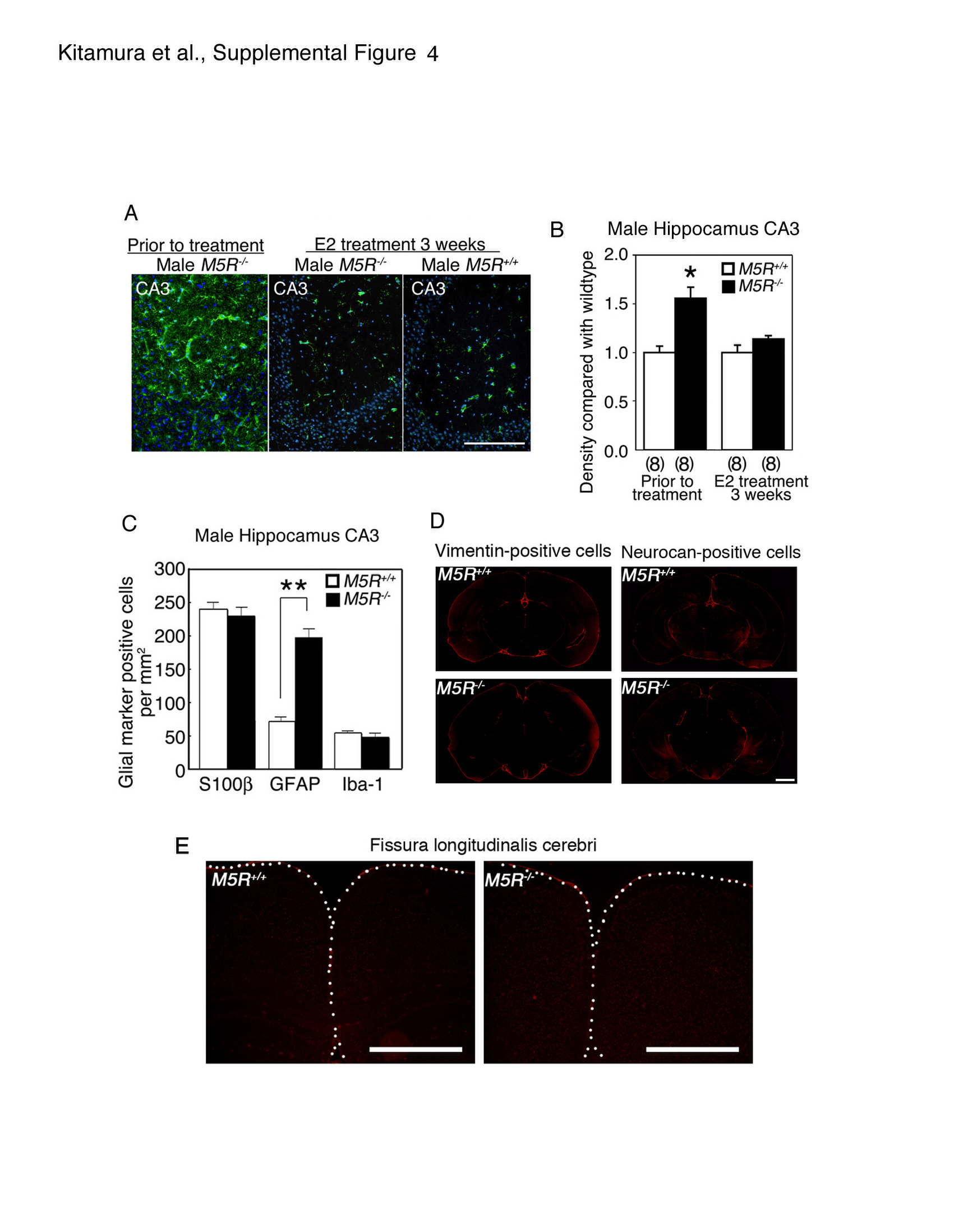

Supplement: Figure S4 — Male M5R−/− mice exhibit astrocyte activation without migration of microglia. Frozen sections of cerebral cortex and hippocampus were prepared from 4-month-old male M5R+/+ and M5R−/− mice. An E2 tablet (0.1 mg/21 days release) was implanted into neck of each mouse. (A) GFAP immunostaining signals in the hippocampal CA3 region from E2-treated male M5R−/− and M5R+/+ mice vs. non-treated male M5R−/− mice. Scale bar = 200 µm. (B) E2 treatment of male M5R−/− mice restored wild-type-like GFAP protein expression levels in the hippocampus, as studied by western blotting analysis. E2-treated male M5R+/+ mice showed similar GFAP protein expression levels as non-treated male M5R+/+ mice. Data are means±SEM (n = 8 per group). *p<0.05 (vs M5R+/+ mice). (C) Male M5R−/− mice showed a significantly increased number of GFAP positive cells in the CA3 region of the hippocampus. However, the number of S100β positive astrocytes, a measure of the total number of astrocytes, remained unchanged. Five hippocampal CA3 sections from 5 animals per group were analyzed. Values are means±SE. **p<0.001. (D) Vimentin and neurocan, markers for injured astrocytes, did not show any increase in immunoreactivity in male M5R−/− mice. Scale bar, 1 mm. (E) Leakage of EB in surrounding blood vessels was not detected in cortex and hippocampus of male M5R−/− and M5R+/+ mice. Scale bar, 1 mm. (11.24 MB TIF) [file pone.0005159.s004.tif]
